# Lipid Signaling in Ocular Neovascularization

**DOI:** 10.3390/ijms21134758

**Published:** 2020-07-04

**Authors:** Ryo Terao, Hiroki Kaneko

**Affiliations:** 1 Department of Ophthalmology, Graduate School of Medicine, The University of Tokyo, Tokyo 113-0033, Japan; 2 Department of Ophthalmology, Nagoya University Graduate School of Medicine, Nagoya 466-8550, Japan; h-kaneko@med.nagoya-u.ac.jp

**Keywords:** growth factors and cytokines, polyunsaturated fatty acid, prostaglandin, glycerophospholipid, lysophosphatidic acid, sphingolipid, sphingosine 1-phosphate, angiogenesis, age-related macular degeneration, diabetic retinopathy

## Abstract

Vasculogenesis and angiogenesis play a crucial role in embryonic development. Pathological neovascularization in ocular tissues can lead to vision-threatening vascular diseases, including proliferative diabetic retinopathy, retinal vein occlusion, retinopathy of prematurity, choroidal neovascularization, and corneal neovascularization. Neovascularization involves various cellular processes and signaling pathways and is regulated by angiogenic factors such as vascular endothelial growth factor (VEGF) and hypoxia-inducible factor (HIF). Modulating these circuits may represent a promising strategy to treat ocular neovascular diseases. Lipid mediators derived from membrane lipids are abundantly present in most tissues and exert a wide range of biological functions by regulating various signaling pathways. In particular, glycerophospholipids, sphingolipids, and polyunsaturated fatty acids exert potent pro-angiogenic or anti-angiogenic effects, according to the findings of numerous preclinical and clinical studies. In this review, we summarize the current knowledge regarding the regulation of ocular neovascularization by lipid mediators and their metabolites. A better understanding of the effects of lipid signaling in neovascularization may provide novel therapeutic strategies to treat ocular neovascular diseases and other human disorders.

## 1. Introduction

Living organisms are composed of various organic compounds, including proteins, carbohydrates, nucleic acids, and lipids. Lipids play crucial roles in numerous cellular processes, acting as cellular structural components and biological barriers and regulating numerous signaling pathways [1,2]. The biological membranes of eukaryotes are amphiphilic sheaths consisting of a lipid bilayer, which acts as a cell barrier [3]. The lipid backbones, head groups, chain length, and position and number of carbon double bonds in fatty acyl chains vary immensely, contributing to the diversity of membranes [4]. Glycerophospholipids, sphingolipids, and sterols are major components of membrane lipids [5]. Additionally, bioactive lipid mediators produced from membrane lipids play pivotal roles in various biological processes and have been implicated in numerous disorders [6,7,8,9,10]. Mounting evidence implies that lipid signaling plays a crucial role in angiogenesis [11,12].

### Signaling in Ocular Neovascularization

Both vasculogenesis and angiogenesis are essential for the formation of vascular networks. Vasculogenesis is the process of blood vessel development by endothelial cells originating from mesoderm-derived progenitor cells [13]. Angiogenesis is the formation of new vessels in response to angiogenic factors; the new vessels are formed by sprouting of endothelial cells in pre-existing vessels [14]. Angiogenesis is profound during embryogenesis but also occurs in adult tissues under certain circumstances, such as wound healing and tumor development [15]. Both vasculogenesis and angiogenesis are involved in neovascularization [16].

Mature blood vessels usually remain quiescent, and angiogenesis does not occur under physiological conditions. However, several stimuli can trigger neovascularization, often leading to pathogenesis. For instance, chronic hypoxia induces the production of angiogenic factors in human tissues, promoting vascular growth from pre-existing vessels [17]. Angiogenesis occurs in several steps. Angiogenic factors trigger endothelial cell sprouting [18], followed by their proliferation and migration and subsequent vessel elongation [19]. A subset of activated endothelial cells become tip cells extending numerous filopodia; tip cells guide the vessel sprout toward angiogenic factors, such as vascular endothelial growth factor (VEGF) and delta-like ligand 4 (DLL4) [20]. Upon Notch signaling, endothelial cells adjacent to tip cells turn to stalk cells [21], which promote lumen formation. The new vessels maturate after the formation of the basement membrane by pericytes and vascular smooth muscle cells [22].

Although angiogenesis is regulated by numerous factors, including fibroblast growth factors (FGF), neuropilins, platelet-derived growth factors (PDGF), and angiopoietins, VEGF is widely considered a master regulator of the process. VEGF gradients in avascular areas guide tip cell migration and induce stalk cell formation, promoting the elongation of new vessels [23]. *Vegf* knockout (-/-) in mice is embryonically lethal around midgestation due to vascular defects [24]. VEGF binding to VEGF receptors (VEGFR1-3) regulates angiogenesis, vasculogenesis, and lymphangiogenesis [25]. Among these receptors, VEGFR1 and VEGFR2 are predominantly expressed in endothelial cells. VEGFR2, rather than VEGFR1, is involved in angiogenesis by regulating endothelial cell proliferation, migration, and viability [26]. Cellular responses to VEGF/VEGFR singling facilitate both physiological and pathological angiogenesis by modulating other bioactive molecules, including endothelial nitric oxide synthase (eNOS), mammalian target of rapamycin (mTOR), and Rho family guanosine triphosphatases (GTPases) [27,28]. In addition to VEGF, hypoxia-inducible factor (HIF) is another critical angiogenic factor. Under hypoxic conditions, HIF-1α translocates into the nucleus and dimerizes with HIF-1β binding to hypoxia response elements (HRE); HIF-1α/ HIF-1β dimers promote angiogenesis by inducing the expression of VEGF, glucose transporter 1 (GLUT1), erythropoietin, and inducible nitric oxide synthase (iNOS) [29]. Interleukin (IL)-8 is a pro-angiogenic cytokine; it activates various molecules, including HIF-1, NF-κB, and signal transducers and activator of transcription 3 (STAT3) via CXC chemokine receptor 1 and 2 [30]. Furthermore, IL-8 transactivates VEGFR2 in endothelial cells, enhancing endothelial permeability and promoting angiogenesis [31]. Vascular endothelial (VE)-cadherin is a component of endothelial junctions, which has been shown to drive angiogenesis. VE-cadherin phosphorylation by VEGF induces endothelial junction disruption and internalization, promoting endothelial cell migration [32]. 

**Figure 1:**
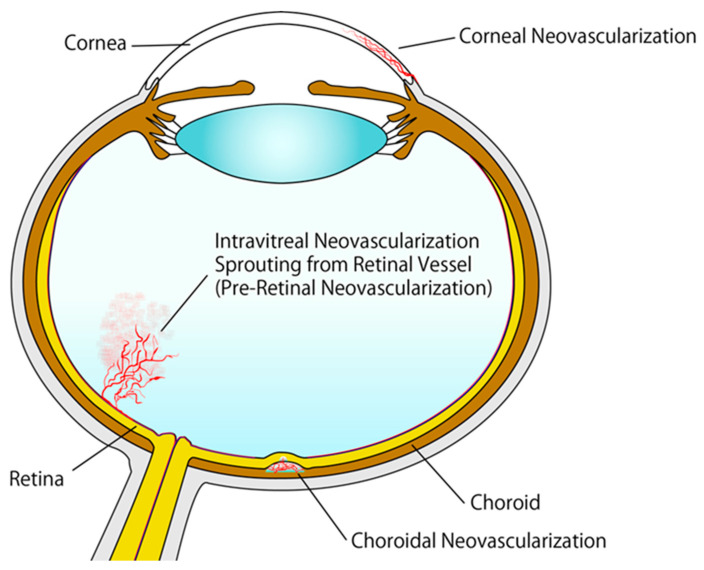
Schematic representation of ocular neovascularization.

The importance of angiogenesis is reflected in the fact that defects in the process have been implicated in numerous disorders, including cardiovascular diseases and cancer, potentially leading to death [33]. Ocular neovascularization is one of the conditions caused by aberrant angiogenesis. Ocular neovascularization is characterized by pathological neovascularization in the retina, choroid, iris, and cornea (Figure 1) [34]. It is observed in individuals with retinal vein occlusion (RVO), retinopathy of prematurity (ROP), diabetic retinopathy, age-related macular degeneration (AMD), neovascular glaucoma, and corneal neovascularization induced by trauma or inflammation [35]. 

**Figure 2:**
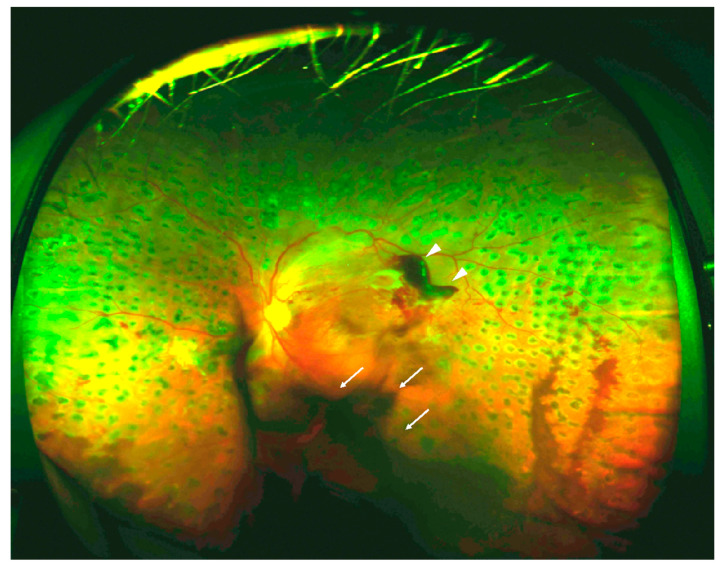
Fundus image of proliferative diabetic retinopathy (PDR) presenting with vitreous (arrow) and preretinal hemorrhage (arrowhead).

Retinal neovascularization and choroidal neovascularization (CNV) are the most common vascular diseases of the eye. Retinal neovascularization diseases, such as RVO, ROP, and diabetic retinopathy, are primarily caused by chronic or severe retinal ischemia [36]. Retinal ischemia induces the production of intravitreal angiogenic factors [37], leading to the proliferation of retinal blood vessels. Pre-retinal neovascularization in the vitreous cavity can cause tractional retinal detachment and vitreous hemorrhage due to increased vascular vulnerability (Figure 2). In severe retinopathy cases, pre-retinal neovascularization can lead to vision loss. Considering its importance in angiogenesis, VEGF is a promising therapeutic target for pathologic retinal neovascularization. Hence, intravitreal injection of VEGF-targeting agents is currently a standard treatment option for patients with visual impairments due to pathologic retinal neovascularization [38,39]. 

AMD is another common condition that can cause visual impairments. It is more prevalent in older people and in developed countries. In AMD, lesions often develop within the macula, the central area of the retina responsible for high-acuity vision. Early-stage AMD manifests as abnormalities in retinal pigment epithelial (RPE) and drusen deposits without impaired visual acuity [40]. Lipoproteins and cholesterol represent major components of soft drusen deposits. Late-stage AMD manifests as exudative AMD or geographic atrophy. CNV is a hallmark of exudative AMD, causing subretinal hemorrhage and vascular leakage (Figure 1). Currently, intravitreal administration of VEGF-targeting agents is the most common treatment for exudative AMD [41,42,43]. 

The cornea is characterized by complete avascularity under physiological conditions to maintain the clarity required for visual acuity [44]. Under pathological conditions, including corneal infection, traumatic injury, ocular surface inflammation, and limbal stem cell deficiency, corneal neovascularization and sprouting from the pericorneal plexus can occur [45,46,47], potentially leading to vision loss. The new vessels can induce corneal opacification and impair vision. Patients with severe corneal neovascularization may require corneal transplantation. Various corneal neovascularization animal models have been established to investigate disease pathology and test novel therapeutics for artificial general neovascularization as well as corneal neovascularization [48]; in most of these models, corneal neovascularization is induced by suture, chemical treatments, and surgical implantation of hydron pellets with reagents containing angiogenic factors [49].

Although numerous efforts have been made to elucidate the mechanisms underlying ocular neovascularization diseases, the role of bioactive lipids remains elusive. In this review, we summarize current knowledge regarding the role of lipid signaling in ocular neovascularization. 

## 2. Glycerophospholipids in Ocular Neovascularization

**Figure 3:**
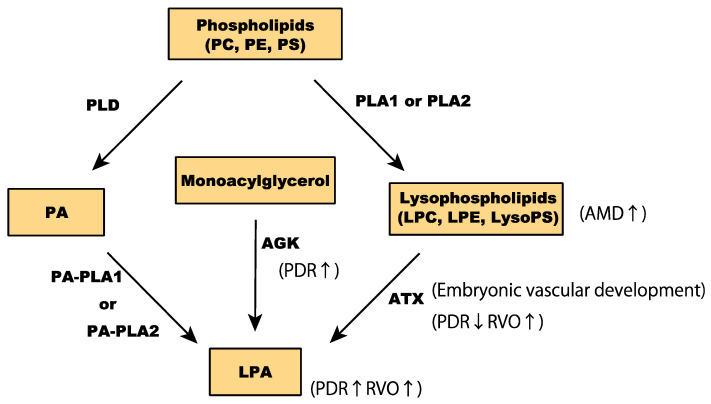
Conversion of glycerophospholipids to LPA (lysophosphatidic acid).

Glycerophospholipids are the most prevalent membrane lipid in mammalian cells. They are composed of a glycerol backbone, two long-chain fatty acids at sn-1 and sn-2 positions of glycerol, and phosphoric acid esterified at sn-3 as a headgroup [50]. The different combinations of headgroups and fatty acids allows for over a thousand glycerophospholipid variants in mammalian cells. Based on their polar headgroups, glycerophospholipids can be classified into phosphatidic acids (PA), phosphatidylcholine (PC), phosphatidylethanolamines (PE), phosphatidylserines (PS), phosphatidylglycerol, and phosphatidylinositol (Figure 3) [51]. 

Lysophospholipids are synthesized from glycerophospholipids by phospholipase A (PLA). PLA_1_ or PLA_2_ enzymatically remove an acyl chain at sn-1 or sn-2 [52]. Among lysophospholipids, lysophosphatidylcholine (LPC) and lysophosphatidylethanolamine (LPE) are the most abundant in mammalian cells [53]. Other lysophospholipids include lysophosphatidic acid (LPA), lysophosphatidylserine (LysoPS), platelet-activating factor, and 2-arachidonoylglycerol, all of which are involved in various cellular responses by acting as signaling molecules [54]. Despite the low intracellular levels of LPA and LysoPS, they regulate important processes, including angiogenesis. 

### 2.1. Lysophosphatidic Acid (LPA)

Mounting evidence implies that LPA plays a crucial role in the pathogenesis of ocular neovascularization. Intracellular LPA is synthesized by glycerophosphate acyltransferase, PA-specific PLA (PA-PLA), and acylglycerol kinase (AGK) [55,56,57], whereas extracellular lysophospholipids are converted to LPA by lysophospholipase D (also known as autotaxin; ATX) [58,59]. LPA is predominantly found in the serum and plasma [60,61].

**Figure 4:**
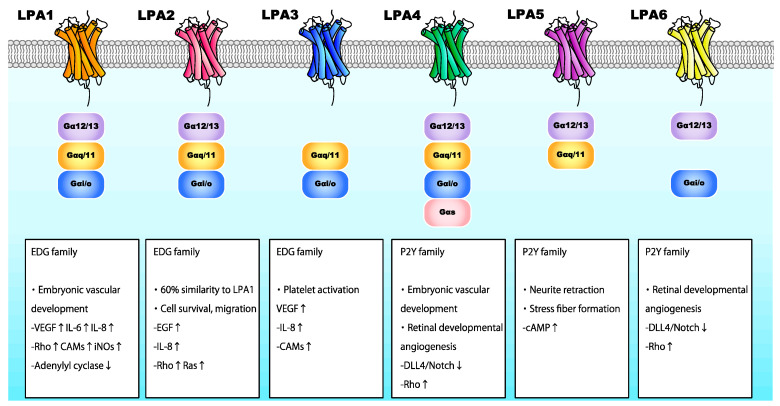
The role of LPA receptors in neovascularization-associated signaling pathways.

LPA is involved in various processes, including angiogenesis, cell proliferation, neurite retraction, and stress fiber formation via LPA receptors [58,62,63,64]. LPA interacts with specific G-protein-coupled receptors found on the cell surface. Six LPA receptors (LPA1-6) have been identified thus far (Figure 4) [65]. LPA1–3 belong to the endothelial differentiation gene family, whereas LPA4–6 belong to the P2Y receptor family [66]. LPA receptors interact with and activate heterotrimeric Gα proteins, namely Gαq/11, Gα12/13, Gαi/o, and Gαs, thereby regulating various cellular functions (Figure 4). Activated G proteins subsequently regulate the activation status of numerous intracellular signaling molecules, including Rho GTPase, mitogen-activated protein kinases (MAPK), protein kinase B (Akt), and phosphoinositide 3-kinase (PI3K) [67]. 

Numerous human gene analysis and preclinical studies have investigated the roles of LPA and LPA receptors [68,69]. These studies have shown that LPA is essential for vascularization [70]. Notably, *Atx*^−/−^ mice were not viable after E10.5 due to severe vascular defects resembling those in *Vegf*^−/−^ mice [71]. *Atx* deletion disrupts the development of vascular networks in the embryo and yolk sac. Allantois explants from *Atx*^−/−^ mice formed vessels, although the lack of ATX and LPA caused endothelial disassembly. These findings imply that the ATX-LPA axis plays a critical role in vascular formation by stabilizing the immature vascular network. Among the LPA receptors, LPA-Gα12/Gα13 is reportedly responsible for this process, as a deficiency thereof results in a similar phenotype to that of *Atx*^−/−^ mice [72]. A previous study using zebrafish revealed the requirement of ATX for vascular development, and that suppression of LPA-LPA1 and/or LPA-LPA4, both of which interact with Gα12/Gα13, was responsible for these vascular defects [73]. Similarly, in the absence of Rho-associated protein kinases (ROCK-1/2), which act downstream of LPA, mice exhibited impaired vascular remodeling in the yolk sac [74]. These findings highlight the vital role of LPA/LPA1 and LPA/LPA4 in vascular development. 

In normal human cells, LPA facilitates angiogenesis by enhancing the production of VEGF, IL-8, MCP-1, and MMP-9 [75,76] in a Gαi/NF-κB-dependent manner, while VEGF upregulation requires the binding of LPA on LPA1 and LPA3 [77]. Moreover, LPA induces HIF-1α expression and nuclear translocation in various cancer cells by activating PI3K/Akt/mTOR and p42/p44 MAPK signaling pathways [78]. Of note, hypoxia or HIF-1α activation enhances LPA-driven cellular responses [79]. Additionally, LPA induces the expression of various cytokines. Notably, LPA promoted IL-6 expression by binding to LPA1 [80], whereas binding to LPA1, LPA2, or LPA3 induced IL-8 expression [81]. The LPA-mediated upregulation of these cytokines was dependent on NF-κB activation by PI3K/Akt and protein kinase C (PKC) signaling pathways. LPA/LPA1 and LPA/LPA3 signaling also upregulated the expression of numerous cell adhesion molecules [82]. LPA binding to LPA1 activated Rho and iNOS in vivo [83,84]. Therefore, it has become evident that LPA regulates angiogenesis in ocular tissues by activating the expression of various angiogenic factors. Nevertheless, the relevance of each LPA receptor in angiogenesis in different tissues remains unclear. 

### 2.2. Lysophosphatidic Acid and Ocular Neovascularization

The biological roles of ATX and LPA in ocular tissues have been partly characterized. ATX is highly expressed in retina [85], and the ATX-encoding gene (*ENPP2*) has been identified as an RPE signature gene [86]. LPA is required for retinogenesis, promoting retinal growth cone collapse [87,88]. In human RPE cells, LPA was shown to regulate barrier integrity [89]. These findings imply involvement of the ATX/LPA axis in retinal function. A recent study assessing the role of LPA and its receptors in angiogenesis during retinal vascular development demonstrated that endothelial cells of mice deficient in LPA4 and LPA6 exhibited reduced filopodia and vessel sprouting, with a reduced number of blood vessels. LPA4/LPA6 coupling with Gα12/Gα13 was also shown to promote retinal angiogenesis, as well as the global vascularization described above, in mice by activating the Gα12/Gα13-Rho-ROCK axis in endothelial cells [90]. Gα12/Gα13-Rho-ROCK pathway activation inhibited DLL4/Notch signaling by activating Yes-associated protein (YAP) and transcriptional co-activator with PDZ-binding motif (TAZ) in endothelial cells at the vascular front. These findings imply that LPA4 and/or LPA6 facilitate tip cell formation and stalk cell proliferation in the retina, promoting angiogenesis (Figure 4). 

Several clinical studies have also investigated the role of LPA in pathological ocular neovascularization. Intravitreal LPA concentrations were significantly elevated in patients with PDR (proliferative diabetic retinopathy) [91]. Intravitreal concentrations of LPA and PA were significantly associated with levels of the inflammatory biomarker vascular cell adhesion molecule-1 (VCAM-1). AGK levels were also higher in PDR patients, implying involvement of the LPA signaling pathway in the pathology of diabetic retinopathy (Figure 3). A subsequent study confirmed the elevated levels of LPA and AGK in the vitreous fluid of PDR patients. On the other hand, ATX levels were significantly lower in PDR patients than in patients without diabetes, implying that AGK-LPA rather than ATX-LPA may be involved in the development and progression of PDR [92]. According to a study investigating LPA expression in RVO, levels of LPA and ATX were significantly higher in vitreous samples from patients with RVO than in samples from patients without RVO. LPA levels were significantly associated with visual acuity impairments and expression levels of MCP-1, VEGF-A, IL-6, and IL-8. Changes in central macular thickness secondary to RVO were also correlated with ATX expression. These findings imply that both LPA and ATX are involved in the pathogenesis of RVO [93]. Nonetheless, the implication of LPA in the pathology of CNV and AMD remains understudied. As LPC metabolism-related markers were higher in the serum of exudative AMD patients [94], it is likely that LPA is also involved in AMD pathology. Future in vivo and in vitro investigations are required to elucidate the biological roles of LPA in pathological ocular neovascularization conditions, in addition to the physiological vascular formation. 

## 3. Sphingolipids in Ocular Neovascularization

**Figure 5:**
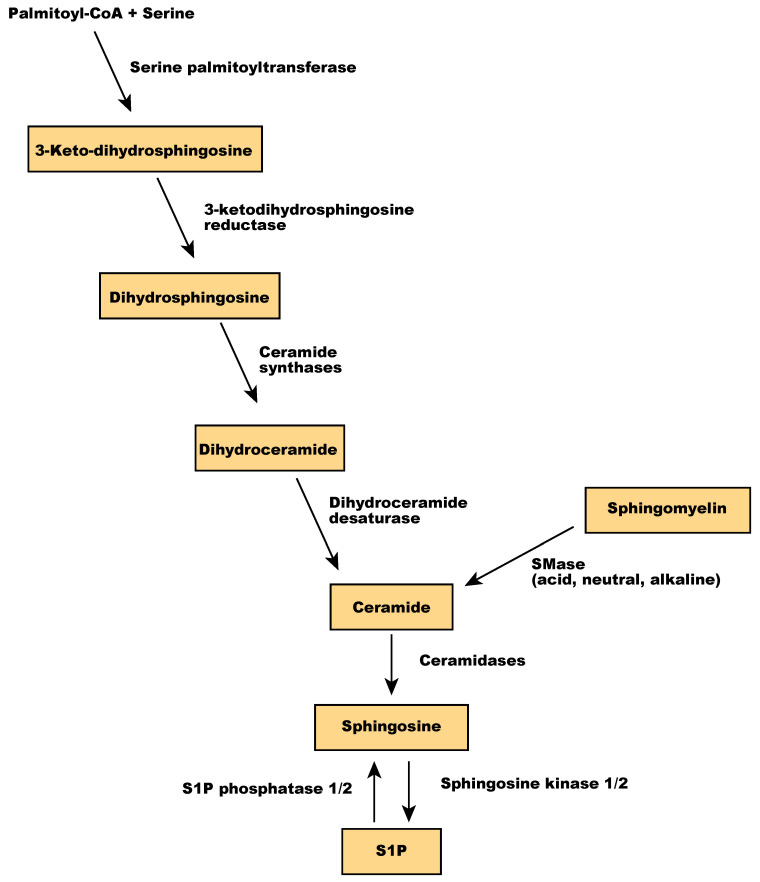
Overview of sphingolipid metabolism.

Sphingolipids are one of the main components of the membrane lipid bilayers in eukaryotic cells. They are derived from palmitoyl-CoA and serine and contain a backbone of sphingoid bases, a fatty acid attached to the long-chain sphingoid base via an amide bond, and a headgroup [95]. In mammalian cells, the headgroup of sphingolipids is a phosphocholine or oligosaccharide. Sphingomyelin, a sphingolipid with phosphocholine as a polar headgroup, is a primary component of membrane microdomains called lipid rafts. Sphingomyelin is hydrolyzed by sphingomyelinases (SMase) into ceramides and phosphocholine [96]. Ceramide regulates various cellular processes, including apoptosis and senescence [97], and is a central player in sphingolipid metabolism because sphingosine is synthesized only from ceramide. Ceramidases, including acid ceramidase, neutral ceramidase, alkaline ceramidase 1 (ACER1), ACER2, and ACER3, catalyze the hydrolysis of ceramide to sphingosine [98], which is then phosphorylated by sphingosine kinases to produce sphingosine 1-phosphate (S1P) (Figure 5). Sphingolipids act as signaling molecules regulating inflammation, cell viability, and migration [99,100]. S1P has been implicated in both physiological and pathological neovascularization, as detailed in the next section. 

### 3.1. Sphingosine 1-Phosphate

**Figure 6:**
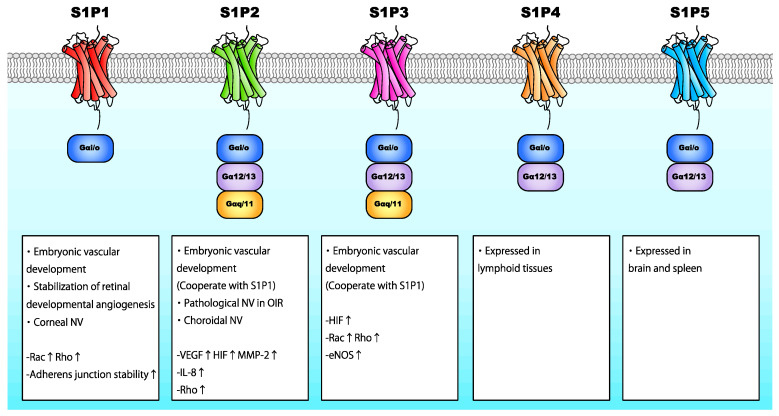
Biological effects of S1P receptors in neovascularization through the interaction with G proteins. S1P exerts various cellular functions by activating different S1P receptors.

S1P is a bioactive lipid consisting of a long-chain sphingoid base and a phosphate polar headgroup. It is synthesized via the ATP-dependent phosphorylation of the hydroxyl group of sphingosine by sphingosine kinase-1 (SphK1) and SphK2 [101]. SphK1 is predominantly found in the cytosol adjacent to the plasma membrane, whereas SphK2 is located in the endoplasmic reticulum, nucleus, and mitochondria [102,103]. S1P interacts with specific G-protein-coupled receptors localized on the cell surface (S1P1-5), initiating autocrine, paracrine, or endocrine signaling. S1P receptors are coupled to Gαi/o, Gαq/11, and Gα12/13 (Figure 6) [104]. S1P1, S1P2, and S1P3 are ubiquitously expressed, whereas S1P4 is primarily expressed in lymphoid tissues, and S1P5 is expressed in the brain and spleen [105,106]. S1P levels are relatively higher in the blood than intracellularly; the concentration of serum S1P in healthy humans is approximately 1 μM [107]. S1P is stored in endothelial cells, erythrocytes, and thrombocytes [108,109].

Similar to LPA, S1P acts as an angiogenic factor, promoting embryonic vascular development. *Sphk1/2*^−/−^ mice were devoid of S1P and embryonically lethal due to profound blood vessel defects between E9.5 and E13.5, leading to cranial hemorrhage and implying that S1P is essential for vascular development [110]. Among S1P receptors, S1P1 is considered the most important for the S1P-mediated effects in vascular development. *S1pr1*-deficient mice exhibited severe vascular smooth muscle defects and hemorrhage, which led to embryonic death [111]. Moreover, the deletion of *S1pr2* and/or *S1pr3* in *S1pr1*^−/−^ mice elicited even more severe vascular maturation defects, leading to lethality. *S1pr1*-*3* triple knockout mice had a reduced number of branches and capillary networks, implying that the interplay of the signaling pathways induced by S1P1, S1P2, and S1P3 is also crucial for vessel development [112]. These reports collectively imply that the S1P/S1P1-3 axis is essential for embryonic vascular development. 

S1P is believed to promote neovascularization through the activation of angiogenic factors (Figure 6). S1P binding to S1P2 promotes VEGF and MMP-2 activation [113]. S1P has been shown to prevent HIF degradation, promoting HIF-1α signaling independently of hypoxia [114]. Additionally, S1P has been shown to induce HIF-1α expression by activating MAPK and PKCβI in a Gαi/o-dependent manner [115]. Conversely, HIF-2α, a HIF-α subunit predominantly expressed in endothelial cells, cardiomyocytes, and glial cells [116], upregulated the expression of SphK1 by binding to *SphK1* promoter [117]. These reports imply that S1P regulates both physiological and pathological angiogenesis by promoting the expression and activation of various angiogenic factors and that hypoxic cellular responses promote S1P expression. Additionally, S1P binding to S1P2 enhanced IL-8 secretion by activating p38 MAPK and extracellular signal-regulated kinase (ERK) 1/2 pathways [118,119]. S1P/S1P1 also modulates endothelial cellular junctions by inducing the translocation of VE-cadherin to adherens junctions and enhancing barrier integrity through Rho and/or Rac activation [120]. On the other hand, the S1P2/Rho/ROCK/PTEN axis as well as VEGF signaling promotes the phosphorylation of VE-cadherin [121]. As different S1P receptors may have opposing biological functions [122], the effects of S1P vary depending on the S1P receptor. 

### 3.2. Sphingosine 1-Phosphate and Ocular Neovascularization

S1P is generated in the retina among other tissues [123], and the essential roles of S1P and S1P receptors in the retinal vascular formation have become apparent. *S1pr1* deficiency in endothelial cells increased the number of tip cells and enhanced filopodia formation. Additionally, *S1pr1* depletion in endothelial cells led to ectopic endothelial hyper-sprouting and subsequent endothelial hyperplasia without pericyte coverage in the retina, resulting in vessel defects and lack of mural cells. The loss of *S1pr1* also impaired VE-cadherin stabilization at endothelial cell–cell junctions, resulting in vascular leakage and lethality [124]. These reports imply an important role for S1P1 in stabilizing sprouting angiogenesis in the retina. Consistently, *S1pr1-3* triple knockout mice exhibited a disorganized retinal vascular endothelium with hyper-sprouting and a lack of vascular endothelial barrier and capillary lumens [125]. Moreover, *S1pr1*-*3* deficient mice had insufficient blood perfusion in the retina and severe vascular structure defects due to impaired endothelial cell specialization. S1P has been shown to regulate vascular maturation partly by suppressing the expression of the transcriptional factor JunB in endothelial cells located behind the vascular front. These findings imply that S1P and S1P receptors (mainly S1P1) are required for endothelial cell specialization and vessel maturation in the retina independently of VEGF. 

In addition to their role in the physiological vascular development, S1P and S1P receptors have been implicated in pathological retinal neovascularization. A study using an oxygen-induced retinopathy (OIR) mouse model demonstrated that *SphK2* overexpression promoted retinal angiogenesis under normoxic conditions [126], reducing the avascular retinal area and exacerbating pathological retinal neovascularization in hypoxia. Conversely, *SphK2*^−/−^ ameliorated retinal neovascularization and decreased the expression of VEGF and angiopoietin, highlighting the crucial role of SphK2/S1P in normal and pathological retinal angiogenesis. Similar to *SphK2*^−/−^ mice, *S1P2*^−/−^ mice lacked intravitreal pathological neovascular tufts, confirming the role of S1P2 in pathological retinal neovascularization [127]. However, in contrast to *SphK2* deletion, *S1pr2* deficiency significantly decreased the avascular retinal area and restored the formation of retinal vasculatures and capillary plexus, partly due to the negative regulation of eNOS. Considering that S1P1 is the most important S1P receptor regulating retinal vessel formation, these results imply that S1P is required for both physiological and pathological retinal angiogenesis and that S1P/S1P2 may be an essential regulator of pathological retinal neovascularization without affecting the retinal vascular morphology. 

Laser-induced CNV and sub-retinal fibrosis induction were alleviated by the intravitreal administration of anti-S1P antibodies [128]. Several humanized anti-S1P monoclonal antibodies repressed CNV in mouse models by inhibiting the IL-8-mediated lymphocyte trafficking [129]. Notably, S1P2 promoted CNV formation by regulating the production of angiogenic factors and inflammatory mediators as well as promoting barrier disruption [130,131]. Although the mechanisms underlying the S1P regulation in pathological neovascularization remain elusive, these findings pinpoint S1P signaling as a promising therapeutic target to suppress pathological retinal and choroidal neovascularization. Unfortunately, a phase II clinical trial found that the intravitreal administration of an anti-S1P antibody could not improve visual impairment in exudative AMD patients [132]. However, there is a possibility that other anti-S1P antibodies or drugs targeting a specific S1P receptor may have the therapeutic effect for exudative AMD. 

Yonetsu et al. [133] evaluated the role of S1P and S1P receptors using a corneal neovascularization rabbit model; they found that an S1P1-3 antagonist suppressed corneal neovascularization. Similarly, the S1P receptor modulator FTY720 attenuated corneal neovascularization and vascular leakage induced by VEGF or S1P [134]. Although it remains unclear which S1P receptor was responsible for these effects, these observations imply that S1P and S1P receptors are also involved in the pathogenesis of corneal neovascularization. 

## 4. The Role of Fatty Acids and Their Metabolites in Ocular Neovascularization

**Figure 7:**
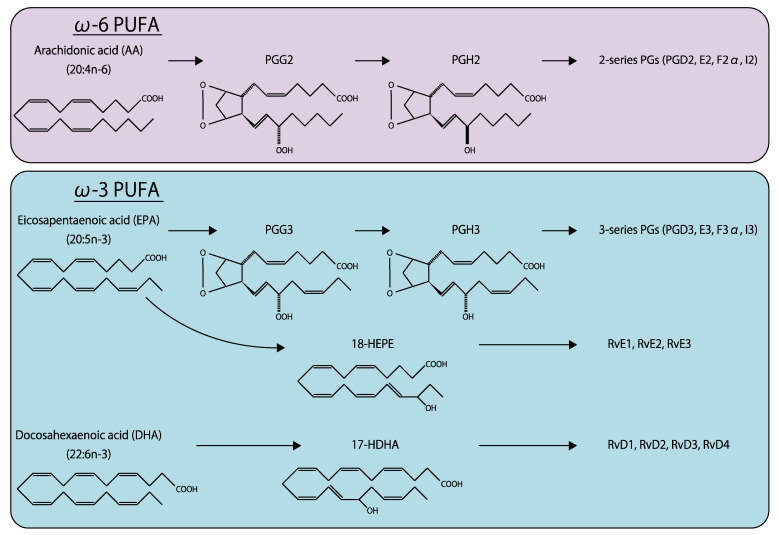
Biochemical structure and metabolism of ω-6 and ω-3 fatty acids.

Eicosanoids are fatty acids synthesized from C20 fatty acids, such as arachidonic acid (AA) and eicosapentaenoic acid (EPA), by fatty acid oxygenases (Figure 7) [135]. Docosanoids are derived from C22 fatty acids, including docosahexaenoic acid (DHA). ω-6 fatty acids, such as prostaglandin (PG), thromboxane (TX), leukotriene, and lipoxin, are produced from AA [136]. These AA metabolites are further metabolized by cyclooxygenase (COX)1 and 2 to produce various bioactive lipids, including PGD_2_, PGE_2_, PGF_2α_, prostacyclin (PGI_2_), and TXA_2_ (also known as 2-series prostanoids), which are generally considered to have proinflammatory effects. In contrast, ω-3 fatty acids generated from EPA, including PGD_3_, PGE_3_, PGF_3__α_, PGI_3_, and TXA_3_ (3-series prostanoids), exert anti-inflammatory effects [137]. Compared with lysophospholipids, the effects of eicosanoids and docosanoids in ocular tissues have been more extensively investigated [138]. 

### 4.1. ω-6 Polyunsaturated Fatty Acids

ω-6 polyunsaturated fatty acids (PUFAs) are considered a pathological angiogenic factor in ocular tissues. Of note, AA or its derivatives promote retinal vascular degeneration, and the inhibition of multiple 2-series prostanoid receptors repressed retinal and choroidal neovascularization in animal models [11,139,140]. 

The biological effects of PGE_2_ have been extensively investigated over many years. It is synthesized by COX from PGH_2_, which also produces PGD_2_, PGF_2α_, PGI_2_, and TXA_2_. PGE_2_ is a potent angiogenic lipid mediator exerting its effects through G protein-coupled receptors (EP1-4) via autocrine and paracrine signaling. EP1 is coupled to Gαq/11, modulating intracellular Ca^2+^ levels [141]. EP2 and EP4 are coupled with Gαs activating adenylate cyclase, which subsequently produces cyclic adenosine monophosphate (cAMP) [142]. In contrast, EP3 is coupled with Gαi/o suppressing adenylate cyclase and promoting Ca^2+^ influx [143]. 

PGE_2_ promotes angiogenesis by enhancing the production of various cytokines, including tumor necrosis factor (TNF)-α, IL-6, basic fibroblast growth factor (bFGF), and VEGF [144,145]. In several tissues, PGE_2_ promotes VEGF and CX3CR1 expression [146,147] partially via PI3K/Akt/mTORC1 pathway activation [148,149]. In HEK-293 cells, PGE_2_ enhanced VEGF and VEGFR1 expression in a Gαi/o-dependent manner [150]. EP4 also promoted VEGF expression [151,152] and has recently emerged as a promising anti-cancer target by suppressing tumor angiogenesis [146,153]. Additionally, PGE_2_ induced TNF-α expression in macrophages [154], as well as enhanced CXCL12 expression, promoting inflammation and angiogenesis [155].

Hypoxia induced the expression of COX-2 and PGE_2_ in retinal Müller cells; PGE_2_ subsequently enhanced VEGF expression, likely via EP2 and/or EP4 [156]. These results imply that PGE_2_ is an important prostanoid associated with retinal neovascularization. Numerous studies have shown that PGE_2_ mediated retinal vascularization in a Gαi/o-dependent manner. Mice lacking EP3 exhibited impaired retinal embryonic angiogenesis due to DLL4/Notch signaling inhibition in endothelial cells [157]. The same study also showed that the endothelial-specific silencing of EP3 attenuated the recruitment of tip cells, implying that EP3 modulates developmental angiogenic processes in endothelial cells. 

EP4 has also been implicated in pathological angiogenesis. PGE_2_/EP4 axis inhibition suppressed pathological neovascularization in OIR and laser-CNV mouse models [158]. The importance of PGE_2_ in corneal neovascularization has also been reported. PGE_2_ levels were elevated in a corneal suture-injury mouse model; PGE_2_ exacerbated corneal neovascularization by promoting chronic inflammatory neovascularization [159]. It is worthy to note that COX2 inhibitors suppressed ocular pathological neovascularization in retina, choroid, and cornea [160,161,162], implicating COX2 is also involved in the pathogenesis of ocular neovascularization as well as PGE_2_. 

### 4.2. ω-3 Polyunsaturated Fatty Acids

In contrast to 2-series prostanoids, 3-series prostanoids have anti-angiogenic and anti-inflammatory effects that protect against several disorders [163]. EPA and DHA found in retinal microvessels are thought to have protective effects against retinal vascular diseases [164]. For instance, ω-3 PUFAs (EPA and DHA) inhibited the expression of TNF-α, IL-1β, VEGF, and cell adhesion molecules in the retina in response to hypoxia or angiogenic factors [165,166]. Similarly, bioactive compounds derived from EPA and DHA, including resolvin E1 (RvE1) and D1 (RvD1), suppressed retinal vascular diseases [167,168]. 

Oral supplementation with ω-3 PUFAs, including EPA and DHA, suppressed retinal obliteration and pathological neovascularization in an OIR mouse model, by inhibiting proinflammatory cytokine secretion in retinal microglia [169]. RvD1 and RvE1 also inhibited neovascularization, implying that ω-3 PUFA supplementation is a promising prevention strategy for retinal vascular diseases. Additionally, peroxisome proliferator-activated receptor (PPAR), whose ligand is ω-3 PUFAs, reportedly has protective effects against pathological retinal and choroidal neovascularization [166,170,171], implicating that PPAR agonists can be considered as a therapeutic option. Furthermore, dietary intake of ω-3 PUFAs may protect against AMD. The prevalence of exudative AMD presenting with CNV was higher in people who did not consume fish, which are enriched in EPA and DHA [172]. Furthermore, ω-3 PUFA intake lowered the twelve-year incidence of AMD [173]. These findings strongly imply that ω-3 PUFAs have a protective effect against AMD. Consistently, ω-3 PUFA supplementation suppressed laser-induced CNV in mice; it also decreased intravitreal concentrations of VEGF-A and alleviated exudative CNV in humans [174,175]. RvD1 and RvE1 attenuated corneal neovascularization induced by herpes simplex virus (HSV)-1 infection, manual suture, or implantation of pellet secreting angiogenic factors. Their anti-angiogenic effects were attributed to their ability to inhibit the infiltration of inflammatory cells and secretion of cytokines, such as VEGF-A, MMP-9, IL-1β, TNF-α [176,177]. The findings of these studies indicate ω-3 PUFAs to be a promising therapeutic option for patients with ocular pathological neovascularization. 

## 5. Future Perspectives

Numerous studies on membrane lipids and their metabolites demonstrate the crucial role of lipid signaling in ocular neovascularization, among other conditions. Glycerophospholipids, sphingolipids, and fatty acids have strong pro-angiogenic or anti-angiogenic effects by activating complex signaling circuits. Future studies are required to elucidate the mechanisms underlying the effects of lipid signaling in ocular neovascularization and other human disorders.

## Abbreviations

AA	arachidonic acid
ACER	alkaline ceramidase
AGK	acylglycerol kinase
Akt	protein kinase B
AMD	age-related macular degeneration
ATX	autotaxin
cAMP	cyclic adenosine monophosphate
CNV	choroidal neovascularization
COX	cyclooxygenase
DHA	docosahexaenoic acid
DLL4	delta-like ligand
eNOS	endothelial nitric oxide synthase
EP	prostaglandin E_2_ receptor
EPA	eicosapentaenoic acid
ERK	extracellular signal-regulated kinase
FGF	fibroblast growth factor
GLUT	glucose transporter
GTPases	guanosine triphosphatases
HIF	hypoxia inducible factor
HSV	herpes simplex virus
HRE	hypoxia response elements
IL	interleukin
iNOS	inducible nitric oxide synthase
LPA	lysophosphatidic acid
LPC	lysophosphatidylcholine
LPE	lysophosphatidylethanolamine
LysoPS	lysophosphatidylserine
MAPK	mitogen-activated protein kinase
mTOR	mammalian target of rapamycin
NV	neovascularization
OIR	oxygen-induced retinopathy
PA	phosphatidic acid
PA-PLA	phosphatidic acid specific phospholipase A
PC	phosphatidyl choline
PDGF	platelet-derived growth factor
PDR	proliferative diabetic retinopathy
PE	phosphatidylethanolamines
PG	prostaglandin
PGI_2_	prostacyclin
PI3K	phosphoinositide 3-kinase
PKC	protein kinase C
PLA	phospholipase A
PLD	phospholipase D
PPAR	peroxisome proliferator-activated receptor
PS	phosphatidylserine
PUFA	polyunsaturated fatty acid
ROCK	Rho-associated protein kinase
ROP	retinopathy of prematurity
RPE	retinal pigment epithelium
Rv	resolvin
RVO	retinal vein occlusion
SMase	sphingomyelinase
STAT	signal transducers and activator of transcription
SphK	sphingosine kinase
S1P	sphingosine 1-phosphate
TAZ	transcriptional co-activator with PDZ-binding motif
TNF	tumor Necrosis Factor
TX	thromboxane
VCAM	vascular cell adhesion molecule
VE	vascular endothelial
VEGF	vascular endothelial growth factor
VEGFR	vascular endothelial growth factor receptor
YAP	yes-associated protein
